# Study on the Evolution of Graphene Defects and the Mechanical and Thermal Properties of GNPs/Cu during CVD Repair Process

**DOI:** 10.3390/ma15010130

**Published:** 2021-12-24

**Authors:** Ziyang Xiu, Boyu Ju, Cungao Duan, Sen Fu, Ningbo Zhang, Yong Mei, Jinming Liu, Yuhan Feng, Wenshu Yang, Pengchao Kang

**Affiliations:** 1 State Key Laboratory of Advanced Welding and Joining, Harbin Institute of Technology, Harbin 150001, China; xiuzy@hit.edu.cn; 2 School of Materials Science and Engineering, Harbin Institute of Technology, Harbin 150001, China; 3 Shanghai Radio Equipment Research Institute, Shanghai 200050, China; lxldxw@126.com (C.D.); nmhyll@126.com (S.F.); 4 Aerospace Research Institute of Materials & Processing Technology, Beijing 100076, China; zhangnbathit@163.com; 5 School of Astronautics, Harbin Institute of Technology, Harbin 150001, China; 6 Defense Engineering of Academy of Military Sciences, PLA Academy of Military Sciences, Beijing 100036, China

**Keywords:** graphene, Cu matrix composite, chemical vapor deposition, defect

## Abstract

Graphene has extremely high theoretical strength and electrothermal properties, and its application to Cu-based composites is expected to achieve a breakthrough in the performance of existing composites. As a nano-reinforced body, graphene often needs a long time of ball milling to make it uniformly dispersed, but the ball milling process inevitably brings damage to the graphene, causing the performance of the composite to deviate from expectations. Therefore, this paper uses CH_4_ as a carbon source to repair graphene through a CVD process to prepare low-damage graphene/Cu composites. The process of graphene defect generation was studied through the ball milling process. The effects of defect content and temperature on the graphene repair process were studied separately. The study found that the graphene defect repair process, the decomposition process of oxygen-containing functional groups, and the deposition process of active C atoms existed simultaneously in the CVD process. When the repair temperature was low, the C atom deposition process and the oxygen-containing functional group decomposition process dominated. In addition, when the repair temperature is high, the graphene defect repair process dominated. 3 wt% graphene/Cu composites were prepared by pressure infiltration, and it was found that the bending strength was increased by 48%, the plasticity was also slightly increased, and the thermal conductivity was increased by 10–40%. This research will help reduce graphene defects, improve the intrinsic properties of graphene, and provide theoretical guidance for the regulation of C defects in composites.

## 1. Introduction

Copper and its alloys have good electrical and thermal conductivity and have important applications in engineering [1,2]. However, the disadvantages of traditional copper materials, such as low strength and poor high-temperature resistance, restrict their industrial application [3,4,5]. The modern industry hopes to obtain a new type of copper material whose strength is far superior to that of traditional copper materials without any significant changes in thermal and electrical conductivity [6,7]. The preparation of composites based on copper is an effective solution. Adding reinforcement to pure copper and its alloys can make up for the lack of performance of copper. Graphene has a series of excellent properties. The elastic modulus can reach 1 TPa, the tensile strength can reach 130 GPa, and the in-plane thermal conductivity can reach 3000~5000 Wm^−1^ K^−1^ at room temperature, which makes graphene a hotspot in materials science since its discovery [8,9,10]. Graphene-enhanced copper-based composites are expected to break through the performance limitations of existing copper materials and obtain higher strength and electrothermal performance [2,11,12].

The high performance of graphene is directly related to its intrinsic structure. The unique two-dimensional structure of graphene has delocalized electrons when intact, making it possess extremely high in-plane electrical conductivity, thermal conductivity, and mechanical properties [13]. However, the structure of graphene is extremely susceptible to damage during material processing. Bartolucc et al. [14] obtained graphene and Al mixed powders by high-energy ball milling, and prepared graphene nanosheets (GNPs)/Al composites by hot pressing. The study found that the Vickers hardness of GNPs/Al composites increased slightly before extrusion, but it was not as good as pure Al after extrusion. At the same time, Graphene Nanoplates (GNPs)/Al have lower tensile strength and strain at break compared to pure Al. The graphene damage was analyzed and characterized by Raman [15,16,17]. Raman characterizes that the D peak of the graphene microchips is higher than the G peak, and both peaks are broadened, indicating that the graphene microchips used in the experiment contain a lot of defects. Rozada et al. [18] found that defects can significantly reduce the intrinsic conductivity of graphene. Shi et al. [19] found in the RGO/polyvinyl alcohol hydrogels system that high-defect materials have lower performance and the loss of defective materials is nearly 50%. The damage to graphene during the material preparation process greatly reduces its intrinsic strength and electrical and thermal conductivity, and at the same time makes it easier to interact with the substrate. The brittle phase Al_4_C_3_ is formed, which reduces the strength of the composites [20,21].

In order to solve the problem of graphene damage, researchers have conducted research from two perspectives: avoiding the introduction of defects during material processing and repairing graphene defects. Short-time high-energy ball milling can greatly reduce the damage to graphene caused by the dispersion process. Yue et al. [22] prepared GNPs/Cu composites by ball milling. Jiang et al. [23] used variable-speed ball milling and modified ball milling processes to prepare GNPs/Al composites. Short-term ball milling greatly reduces the damage of graphene and retains the structural integrity of graphene to the greatest extent, which is conducive to the strengthening of its two-dimensional reinforcement. The tensile strength of the prepared composites is 30–50% higher than that of the matrix.

By reducing the speed of the ball mill and shortening the time of the ball milling, serious damage to the graphene caused by the ball milling process can be avoided. However, short-term ball milling still inevitably introduces a small amount of defects to the graphene. In order to further reduce the defect content of graphene, other methods are used to repair the defects of low-damage ball-milled dispersed graphene. At present, the graphene repair methods reported mainly include high temperature graphitization repair [18,24], Chemical Vapor Deposition (CVD) repair [25,26,27], doping repair [28,29,30], liquid repair [31,32] and so on. Each of the above methods has its own advantages. High-temperature graphitization repair requires the material to be heat-treated for a long time at 2000 °C to cause spontaneous diffusion of atoms to achieve graphene repair. Rozada et al. [18] obtained graphene with very low defect content by annealing the reduced graphene oxide film at high temperature, and the conductivity can reach 5.77 × 10^5^ S/m. It is much higher than RGO (7.96 × 10^3^ S/m) which has only undergone chemical reduction. High-temperature graphitization has the best effect, but it is difficult to apply to metal matrix composite systems. Liquid repair and doping repair work by in-situ self-generation or diffusion into the graphene lattice under liquid conditions to prepare carbon atom-filled or heteroatom-doped graphene structures. It is suitable for liquid phase dispersion of graphene while realizing defect control. However, the repair effect is not obvious enough, and the improvement of material performance is small.

CVD repair mainly uses free radicals generated by the decomposition of carbon-containing gas as a C source at high temperatures to fill and repair graphene defects spontaneously. Zhu et al. [25] used CH_4_ plasma to repair defects in graphene. The study found that the Hall mobility of graphene increases significantly with the increase in the degree of repair, from 1 cm/(V·S) to 52 cm/(V·S), while the resistivity decreases significantly. Grimm et al. [27] used isopropanol as a carbon source to repair graphene defects through CVD. The defect density is greatly reduced, and the average defect spacing of graphene has increased from 2–3 nm to 10–12 nm. Kim et al. [28] used CVD technology to prepare hollow graphene spheres and repaired graphene oxide. The graphene ball does not fill up the defects so that the conductivity of the graphene is increased to 1.8 × 10^4^ S/m, and the agglomeration between graphene layers is avoided, which effectively increases the specific surface area. However, in current research, CVD is mainly used for defect repair of graphene powder. After the graphene and metal powder is dispersed by ball milling, the method and extent of CVD repairing graphene have not been studied.

In this work, pure copper was selected as the matrix and GNPs as the reinforcement. GNPs/Cu composite powder was prepared by high-energy ball milling, and the defects of GNPs during the ball milling process were repaired by the CVD method. The graphene defects were characterized by Raman Spectrum (Raman) and X-ray Photoelectron Spectroscopy (XPS), the repair effect of CVD on graphene defects was studied, and the best defect repair process was explored. The powder is sintered into a composite by Spark Plasma Sintering (SPS). By comparing the mechanical properties, thermal conductivity, and microstructure of the composites with or without repair, the effect of defect repair on the performance is studied, which provides theoretical guidance for the preparation of low-damage GNPs/Cu composites.

## 2. Materials and Methods

**Figure 1:**
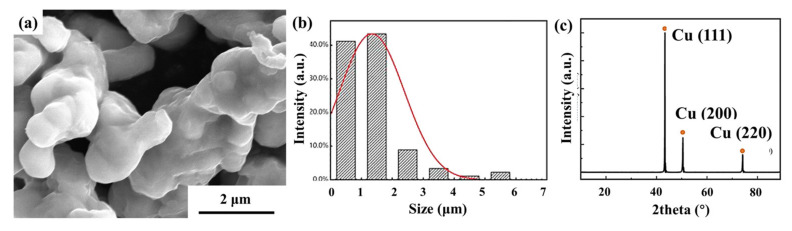
Original Cu powder morphology, particle size distribution and XRD characterization. (**a**) SEM of Cu powder, (**b**) particle size distribution, (**c**) XRD characterization of Cu powder.

The pure Cu powder used in this study was purchased from Beijing Xingrongyuan Co., Ltd., Beijing, China, with a purity of 99.9% and an initial particle size of about 3 μm. The characterization results are shown in Figure 1.

**Figure 2:**
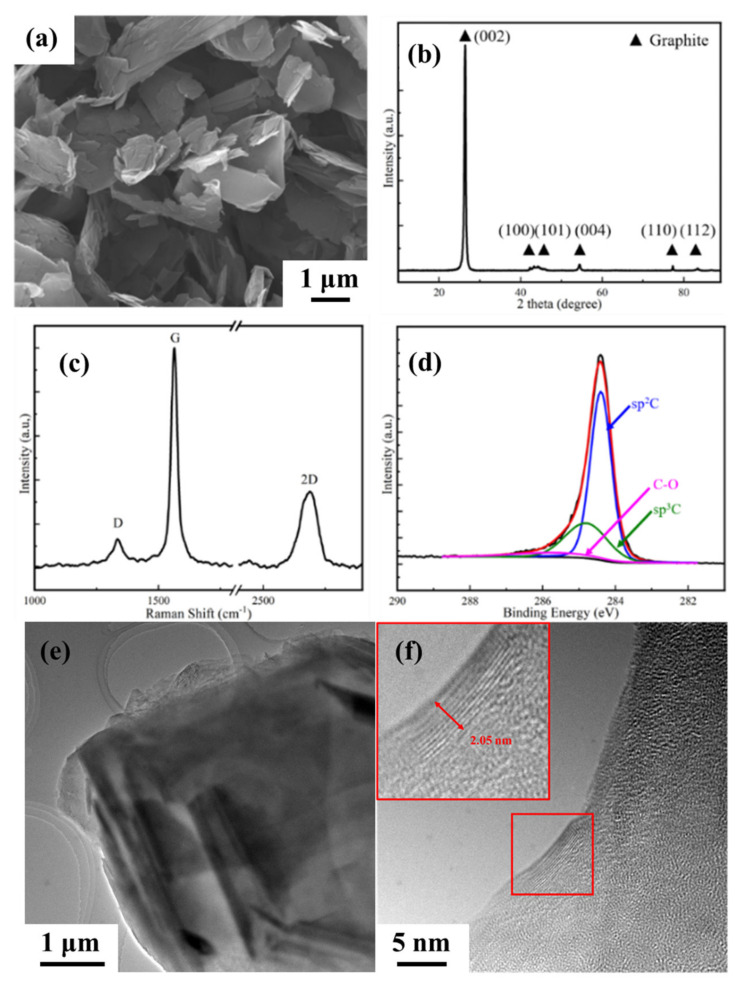
Surface morphology and microstructure characterization of original graphene. (**a**) SEM characterization of graphene, (**b**) XRD characterization result of graphene, (**c**) Raman spectrum characterization of graphene; (**d**) XPS characterization of graphene; (**e**) Bright field image characterization of graphene; (**f**) HRTEM characterization of graphene.

The graphene (supplied by the Sixth Element Changzhou Materials Technology Co., Ltd., Changzhou, China) used in this experiment was prepared by the exfoliation method, and its Raman spectrum test results and XPS test results are shown in Figure 2. It can be seen from SEM that the graphene surface is flat, with fewer wrinkles, a larger sheet diameter, and the overall structure is complete. The Raman spectroscopic characterization results show that the intensity ratio of the D peak to the G peak of graphene is *I_D_*/*I_G_* = 0.19, and the value of *I_D_*/*I_G_* indicates the defect content of graphene. The graphene used in this experiment has fewer defects. The original graphene was subsequently characterized by XPS. The XPS results show that the GNPs C atoms are mainly sp^3^ C, with a small amount of sp^2^ C defects and some oxygen-containing functional group defects. The ratio of the peak area of sp^3^ C atoms to the total area can reflect the content of graphene lattice defects. From the figure, it can be seen that the defect content of the original graphene is about 30%, and the content of functional group defects is about 8%. The TEM characterization of graphene is shown in Figure 2e,f. Layered features can be seen at the edge of graphene, with a thickness of 2.05 nm. The single-layer graphene layer spacing is 0.34 nm, so the number of graphene layers shown in the figure is about 6 layers.

High-energy ball milling is a commonly used method to introduce graphene into Cu powders. This method is easy to operate and has a good mixing effect. However, the impact and shear of the graphene during the ball milling process will also destroy the integrity of the graphene structure. In this study, a planetary ball mill (YXQM-4L, supplied by Miqi Equipment Co., Ltd., Changsha, China) was used to disperse the graphene, and the ball-milling speed of 250 r/min was used to ensure the uniform dispersion. 1.5 g of graphene, 48.5 g of Cu powder and 500 g of ZrO_2_ grinding balls were added to each ball milling jar (the ball-powder ratio is 10:1). In addition, 500 g ethanol was added as a grinding aid. The ball mill selected a speed of 250 r/min, and the ball mill stopped for 25 min every 5 min to prevent the danger of overheating. The graphene and Cu were ball milled for 120 min to make them uniformly dispersed. After ball milling, the graphene-Cu mixed slurry was separated through a screen and dried at 70 °C for 24 h to obtain graphene-Cu mixed powder. The chemical state of graphene after ball milling was characterized by Raman and XPS.

In order to reduce the damage of graphene, CVD is used to repair graphene defects. The CVD process is carried out in a pipe furnace (supplied by Kejing Equipment Co., Ltd., Hefei, China). Methane (CH_4_) was chosen as the carbon source and argon (Ar) was chosen as the shielding gas. The ratio of Ar to CH_4_ is 10:1. In the experiment, 800 °C, 900 °C and 1000 °C were selected to treat the powder to characterize the evolution behavior of graphene defects at different temperatures. The gas flow rate is 20 sccm (stand mL/min), the pressure is 280 Pa, and the reaction continues for 30 min. After cooling in the furnace, the product was taken out for subsequent characterization and experiments.

The mixed powder of GNPs and Cu obtained by ball milling was sintered by the SPS process. The mixed powder of GNPs and Cu was put into a mold with a diameter of 40 mm and pressed into a preform under a pressure of 40 MPa, and the pressure was maintained for 5 min. Then the mold with the preform was put into the SPS equipment, heated to 1000 °C at 5 °C/min, and kept at high temperature for 10 min. The current-to-time ratio (ton:toff) during sintering was 2:1. After the sintering is completed, the sample is cooled to room temperature in the mold with the furnace. Subsequent structural characterization and performance testing of composites.

Morphologies of the GNPs/Cu powders and the composites were observed by FEI Quanta 200FEG (supplied by Thermo Fisher Scientific Co., Ltd., Agawam, MA, USA). Raman analysis was performed on a JY-HR800 laser Raman spectrometer (supplied by HORIBA Ltd., Paris, France) using a 532 nm solid-state laser as an excitation source. X-ray Photoelectron Spectroscopy was performed on ESCALAB 250Xi (supplied by Thermo Scientific, London, UK) spectrometer with a monochromatic Al Ka radiation source at 15 kV and 150 W. Tensile samples with dimensions represented in our previous work have been tested on Instron 5569 universal electrical tensile testing machine (supplied by Instron Ltd., Bostion, MA, USA) with a cross-head speed of 0.5 mm/min. All the tensile tests have been performed on at least four samples to improve the statistical significance of the results. The LFA467 laser thermal analyzer (supplied by NETZSCH-Gerätebau GmbH Ltd., Bavaria, Germany) was used to characterize the thermal diffusivity of the GNPs/Cu composites, and the test temperature was 25 °C.

## 3. Results and Discussion

### 3.1. The Influence of Ball Milling on GNPs Defects

**Figure 3:**
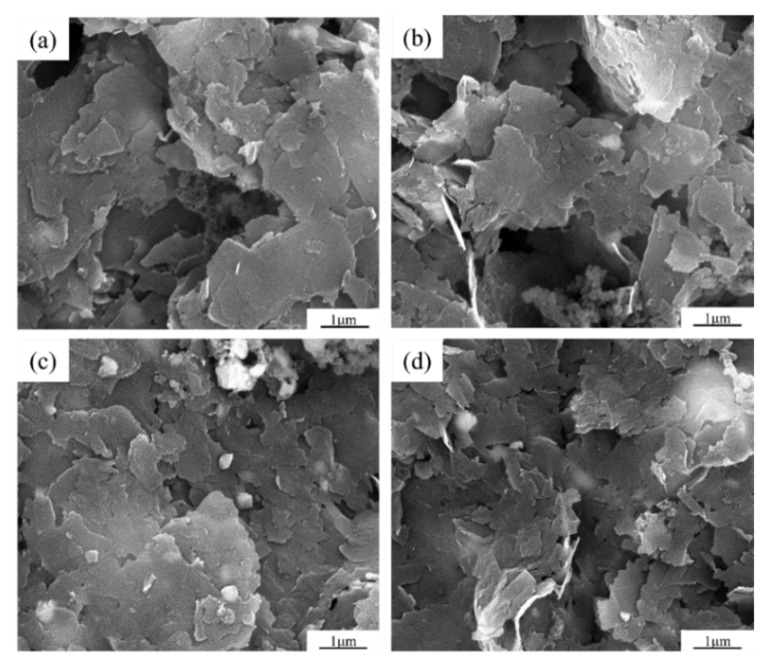
10 SEM photos of graphene after different ball milling time. (**a**) 1 h, (**b**) 2 h, (**c**) 5 h, (**d**) 10 h.

**Figure 4:**
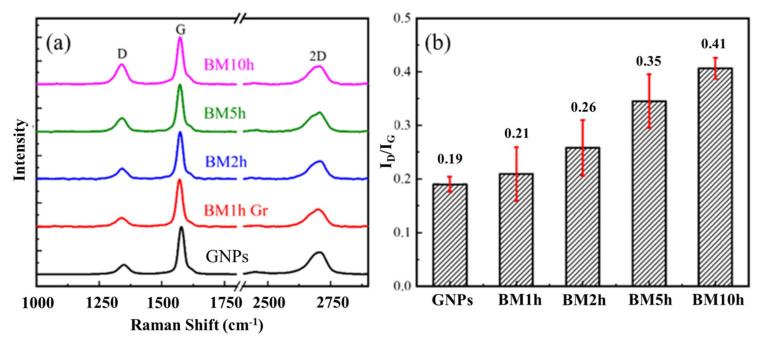
The influence of different ball milling time on graphene defect content. (**a**) Raman spectrum test results of graphene, (**b**) Raman spectrum *I_D_*/*I_G_* statistics of graphene.

To study the ability to which CVD can repair GNPs defects, it is necessary to prepare GNPs with different defect contents. In order to obtain graphene with different defect content, four different ball milling times were designed, namely BM1h, BM2h, BM5h, and BM10h. Figure 3 shows the SEM pictures after different ball-milling times. It can be seen that when the milling time is 1 h and 2 h, the size and morphology of most graphene sheets do not change significantly compared with the raw material graphene. With the prolongation of the milling time, more fragmented graphene appeared, indicating that during the milling process, the graphene was structurally damaged under the impact and shear of the milling ball and the powder. After that, the graphene with different ball-milling times was characterized by Raman spectroscopy, and the results are shown in Figure 4.

The degree of damage of GNPs was characterized by Raman. The intensity of the D peak (*I_D_*) and the G peak (*I_G_*) is generally considered to be an apparent manifestation of the defect content of graphene. This is because when the defect content does not exceed a certain ratio, *I_D_*/*I_G_* is proportional to the average distance between the two defects on the graphene [33], that is:

(1) $$\frac{I_{D}}{I_{G}}=\frac{C_{\lambda }}{L_{d}^{2}}$$

where in *I_D_* and *I_G_* are the intensity of peak D and peak G in the Raman spectrum, respectively; *C_λ_* is a constant related to the wavelength of the test laser; *L_d_* is the average distance between two defects on the graphene.

By characterizing the Raman spectra of graphene with different ball milling times, it can be found that the *I_D_*/*I_G_* gradually increases, reaching the maximum when the milling time is the longest. It shows that with the increase of ball milling time, the defect content in graphene gradually increases. This is also the same as the previous SEM observation results.

**Figure 5:**
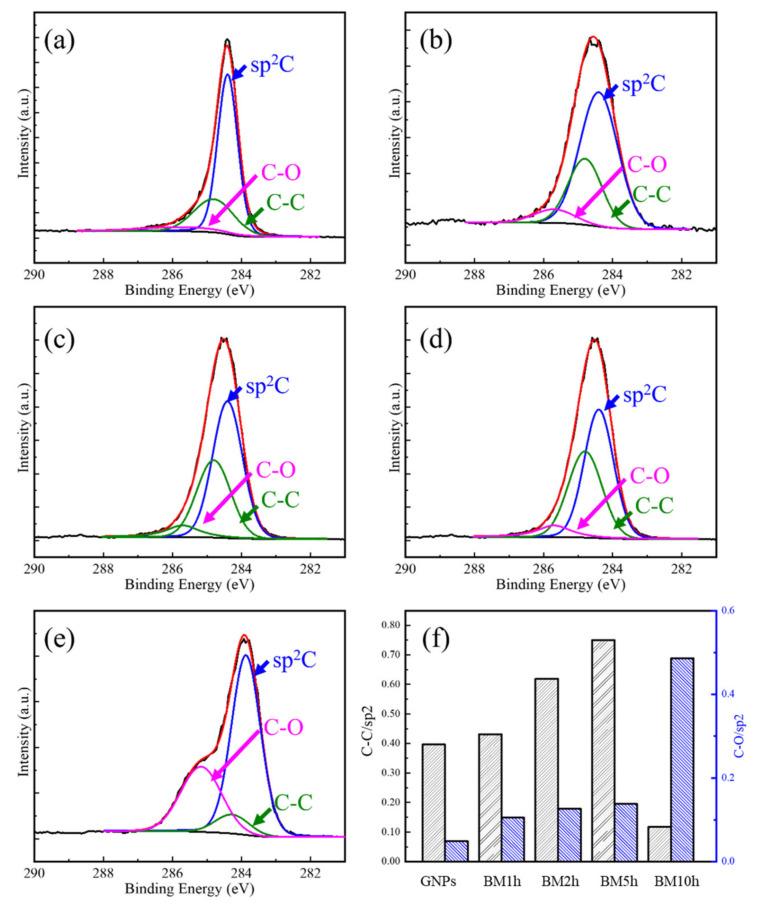
XPS characterization of C1s orbital binding energy of GNPs. (**a**) Initial GNPs, (**b**) BM1h, (**c**) BM2h, (**d**) BM5h, (**e**) BM10h, (**f**) the ratio of the C–C peak area and sp^2^ peak area (C–C/sp^2^) and the C–O peak area and sp^2^ peak area (C–O/sp^2^) of GNPs at different ball-milling time.

XPS characterization of GNPs with different milling time was carried out. The content of atoms in different chemical states in the material was quantitatively evaluated by the peak area, and the results are shown in Figure 5.

It can be seen from Figure 5 that three structures generate XPS signals in the initial GNPs, which are sp^2^ hybridized C atoms, C–C bonds, and C–O groups. The C atom in the sp^2^ hybrid state corresponds to the complete six-membered ring structure in graphene, and both C–C and C–O are sp^3^ type defects. Among them, the content of C–O groups is small and the peak is not obvious, indicating that the graphene has not been severely oxidized, and the defects in the initial GNPs are mainly C–C bonds. After ball milling, an increase in the proportion of the area occupied by the C–C bond and C–O group peak can be observed clearly. When the milling time is 5 h and before, the two increase at the same time. When the milling time continues to be extended, after 10 h, the proportion of C–C decreases sharply while the proportion of C–O increases significantly. This may be due to the O_2_ oxidation of C–C at the edges or defects formed by the destruction of the GNPs structure during the ball milling process.

### 3.2. Study on the Evolution Behavior of GNPs Defects during CVD

The ball milling process inevitably causes damage to graphene. In order to reduce the content of hole defects in graphene, CVD is used to process the ball-milled powder. In this paper, CH_4_ is selected as the carbon source, and the repair effect of CVD on graphene with different defect contents under different temperatures is studied.

#### 3.2.1. The Influence of GNPs Defect Content

**Figure 6:**
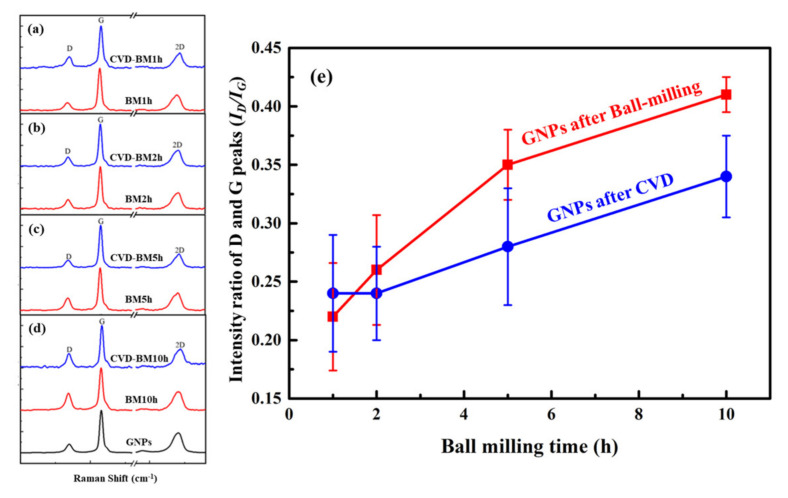
Raman characterization of GNPs after ball milling and CVD repair. (**a**–**d**) Raman characterization curve of GNPs after ball milling for 1 h, 2 h, 5 h, 10 h and CVD repair; (**e**) The *I_D_*/*I_G_* variation law of GNPs at different milling time.

In this study, four different GNPs with different initial defect content were prepared by changing the milling time, and the repair effects were compared under the CVD repair process at 1000 °C. The result is shown in Figure 6.

It can be seen that after repairing at 1000 °C, the content of defects generated in a short time has a slight downward trend. This may be due to more vacancy defects in GNPs, which are repaired by active C atoms at high temperatures. When the ball milling time is 5 h, the defect content in GNPs has increased significantly. At this time, more vacancies and sp^3^ hybridization defects have appeared in GNPs. After being repaired, the defect content has dropped significantly, indicating that the repair effect has a significant effect on these two types of defects. However, the degree of dispersion of the data is large, indicating that the repair effect is not uniform. Continue to extend the milling time. Under the milling time of 10 h, the subsequent increase in defect content is mainly due to vacancies and sp^3^ hybrid defects. After repairing at 1000 °C, the defect content has decreased more obviously, and the statistical distribution of *I_D_*/*I_G_* is more uniform, indicating that a better and more consistent repair effect has been achieved. Therefore, graphene ball milled for 10 h was used as the object of restoration research in the follow-up research. BM1h has fewer defects introduced by ball milling, so it was also studied as a control.

#### 3.2.2. The Influence of CVD Repair Temperature

Temperature is an important factor affecting the repair process of GNPs. Temperature affects the decomposition behavior of CH_4_ and the diffusion behavior of C atoms. Temperature also has a greater impact on the interaction between C atoms and GNPs, and is an important factor affecting the GNPs repair effect. In this study, the effects of the CVD process on the repair effect of graphene at different temperatures are studied. BM10h and BM1h were selected as the raw material to study the effect of repair CVD time on the repair process.

**Figure 7:**
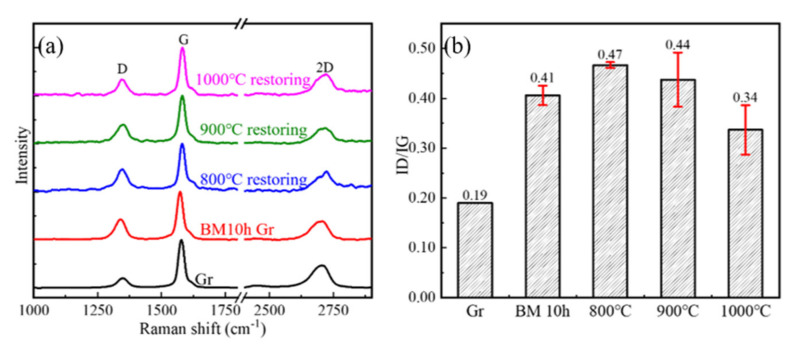
The Raman spectrum and *I_D_*/*I_G_* statistics of BM10h GNPs repaired at different temperatures in CVD process. (**a**) Raman spectrum, (**b**) *I_D_*/*I_G_* statistics.

**Figure 8:**
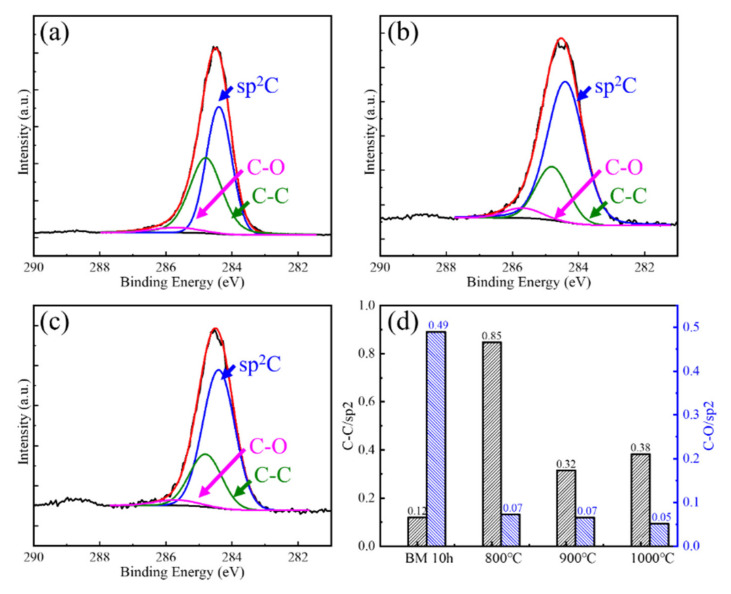
XPS characterization and fitting results of CVD repaired BM10h at different temperatures. (**a**) 800 °C, (**b**) 900 °C, (**c**) 1000 °C, (**d**) C–C/sp^2^ and C–O/sp^2^ change with CVD temperature.

The defect evolution behaviors of BM10h GNPs at different CVD temperatures are shown in Figure 7 and Figure 8. In Figure 8, the defects of GNPs showed a trend of rising first and then falling as the temperature increased. Combined with the characterization results of XPS, the defect status in GNPs was analyzed. It can be seen that in BM10h, the defects of graphene are mainly C–C defects and C–O defects.

At 800 °C, Raman shows that the defect content of GNPs increases. XPS results show that the C–O defects of graphene are greatly reduced at 800 °C, indicating that the oxygen-containing functional group defects of graphene are repaired during the CVD process. However, the C–C/sp^2^ content increased, which was attributed to the deposition of C atoms produced by the decomposition of CH_4_ on the surface of GNPs. As the CVD temperature further rises to 900–1000 °C, the C–O bond content further decreases, and at the same time the C–C bond content also greatly decreases. This is because C atoms begin to diffuse to a large extent after the temperature rises, spontaneously repairing the hole defects of GNPs, resulting in a substantial decrease in C–C/sp^2^.

**Figure 9:**
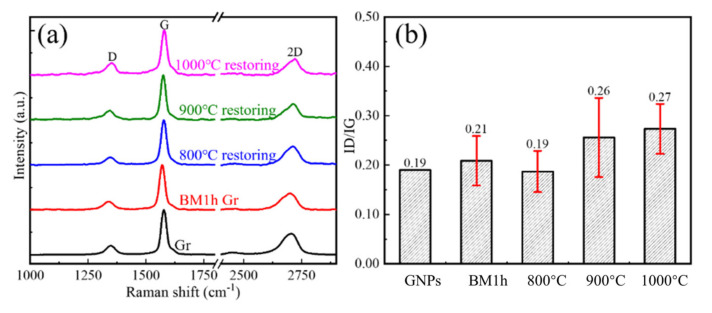
The Raman spectrum and *I_D_*/*I_G_* statistics of BM1h GNPs repaired at different temperatures in CVD process. (**a**) Raman spectrum, (**b**) *I_D_*/*I_G_* statistics.

**Figure 10:**
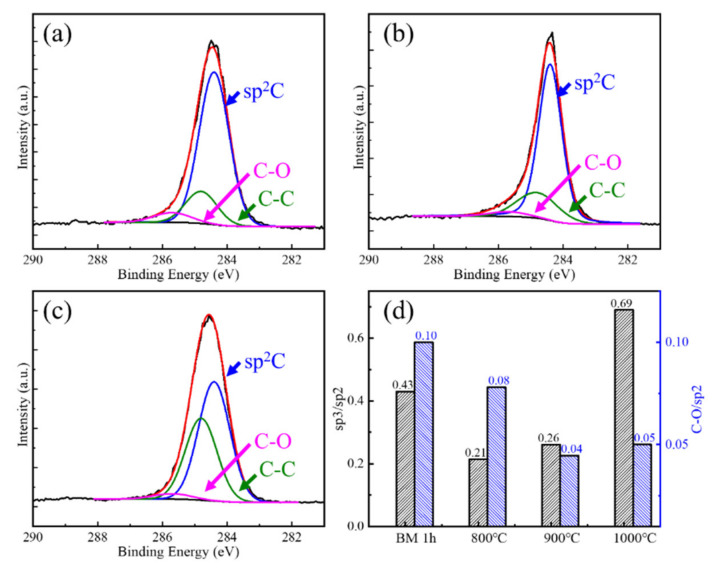
XPS characterization and fitting results of CVD repaired BM10h at different temperatures. (**a**) 800 °C, (**b**) 900 °C, (**c**) 1000 °C, (**d**) sp^3^/sp^2^ and C–O/sp^2^ change with CVD temperature.

The law of BM1h GNPs defects with CVD temperature variation is characterized by Raman and XPS, as shown in Figure 9 and Figure 10, respectively. At 800 °C, GNPs achieved a good repair effect, and their defect content was close to that of the original graphene. As the repair temperature increases, the defect content of GNPs also increases. At 900 °C and 1000 °C, the defect content increased slightly. In general, the defect content after repair shows a trend of first decreasing and then increasing.

XPS characterization of the interatomic binding energies of repaired GNPs at 800 °C, 900 °C, and 1000 °C was carried out, and the change of C atom content in different existing states was semi-quantitatively characterized. The result is shown in Figure 8. It can be seen that as the treatment temperature increases, the content of C–O bonds decreases to the same level as the initial GNPs, which shows that high temperature is beneficial to the complete removal of C–O.

The content of C–C bonds decreased significantly at 800 °C and 900 °C, which was mainly related to the repair of vacancies. It increased significantly at 1000 °C, indicating that more high-defect graphene was produced at this time. This phenomenon was not observed when it was low. This may be related to the difference in the rate of graphene generated by CVD at different temperatures. At low temperatures, the decomposition of CH_4_ is slower and the content of active C atoms is less, so the yield of graphene is also lower; CH_4_ decomposes faster at high temperatures, and the rate of GNPs generation Faster, higher content, and the formation of graphene with a large number of defects caused an increase in the C-C bond content and the overall defect content of GNPs.

### 3.3. Research on the Mechanism of GNPs Defect Repair

Through the previous analysis, it can be known that when the milling time is short, the defects generated are mainly vacancies, C–C bonds, and edges, and oxidation is not obvious. When the ball milling time is longer, the defect C–O bond is mainly generated, and obvious oxidation occurs. It is reported in the literature that C–O is not stable at high temperatures, and O can easily further react with C to form CO_2_, which takes away the C atoms in GNPs in the form of gas. Since GNPs are always in a protective atmosphere, the newly generated vacancy defects will not be oxidized again, so the C–O content is greatly reduced after the repair treatment, which is also consistent with the characterization results of XPS. At the same time, it can be observed that after the repair treatment, the content of C–C atoms increased. This may be due to the active C atoms decomposed by CH_4_ during the repair process nucleating and growing on the surface of the Cu sheet, generating new high defects. Caused by the content of graphene.

**Figure 11:**
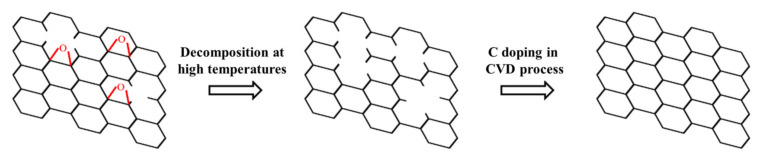
Schematic diagram of the repair process of BM introducing defect graphene.

The repair of graphene is related to the decomposition of C–O functional groups at high temperatures. The repair process is shown in Figure 11. First, during the heating process, the C–O functional groups contained in the in-plane and edges of GNPs decompose, and C atoms are taken away during the decomposition process, and vacancy defects are generated at the functional groups. After passing in CH_4_ and H_2_, under the catalysis of Cu, CH_4_ is decomposed into active C atoms and H atoms. Among them, the active C atoms are easy to combine with the defects, fill the vacancies, repair the defects of GNPs, and form complete graphene. Six-membered ring structure. In this process, H atoms are likely to have an etching effect on graphene, especially the edge area, taking away C atoms, and at the same time, the newly generated active C atoms supplement the area etched by H and promote the growth of graphene sheets. It is worth noting that, according to Zhu et al. [25], the etching of H to GNPs on the deposition of C on the graphene surface also has a competitive relationship. When the temperature is low, the etching effect is dominant, which makes the thickness of GNPs thinner and the light transmittance increases; when the temperature is higher, the deposition effect dominates, which increases the thickness of GNPs and reduces the light transmittance. At the same time, active C atoms can also nucleate and grow on the Cu surface under the action of high temperatures, forming new graphene.

In the process of repairing GNPs with a ball milling time of 1 h, the decomposition of C–O and the formation of vacancies occurred at a temperature of 800 °C. Although there is the influence of the deposition of C atoms, the temperature is low at this time, the content of C atoms in the atmosphere is not high, and the deposition is not obvious. Due to the shorter ball milling time, the degree of oxidation in graphene is lighter, there are fewer vacancies generated during the decomposition of C–O functional groups, and the repair of defects by C atoms is easier, which reduces the defect content. When the temperature continues to rise, the deposition of C atoms dominates, and the newly formed graphene has a greater influence on the Raman spectral signal, which causes the defect content of GNPs to increase. In the process of repairing GNPs with a ball milling time of 10h, since the oxidation degree of GNPs is relatively serious at this time, a large number of vacancy defects are generated during the decomposition of C–O. At a temperature of 800 °C, the speed of repairing defects by active C atoms is not as fast as that of CO decomposition to produce new vacancy defects. At the same time, it is affected by the newly generated high defect content GNPs, and the overall defect content of GNPs further increases; in addition, due to CO decomposes, and the out-of-plane atoms connected to the C atoms on the GNPs surface are reduced, resulting in a reduction in the defect content caused by sp^3^ hybridization. When the temperature rises, the decomposition rate of CH_4_ increases, and the ability to fill defects is enhanced, resulting in a significant decrease in the content of defects.

### 3.4. Performance Test of Repaired Graphene/Cu Composite

**Figure 12:**
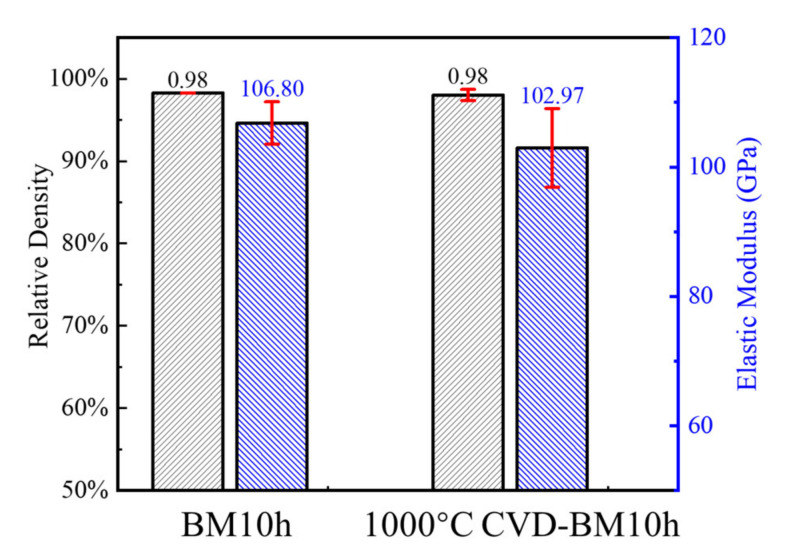
Density and elastic modulus of composites made by BM10h and 1000 °C CVD-BM10h.

The composites were prepared with GNPs-Cu powder ball milled for 10 h (BM10h) and ball milled powder repaired by CVD at 1000 °C (1000 °C CVD-BM10h), which have the most defects and the most obvious graphene repair effect. The density and elastic modulus of the composite material were characterized, and the results were shown in Figure 12. It can be seen that the density of the sintered composite is relatively high, and the density of the GNPs/Cu composite after the CVD defect repair process and the unrepaired GNPs/Cu composite are both 98%, showing good compactness. It shows that in the sintering process, the powder inside the composites achieves a better bonding effect. The elastic modulus of the two composite materials is about 105 GPa, which is slightly lower than that of pure copper, which may be caused by the existence of voids in the composites.

**Figure 13:**
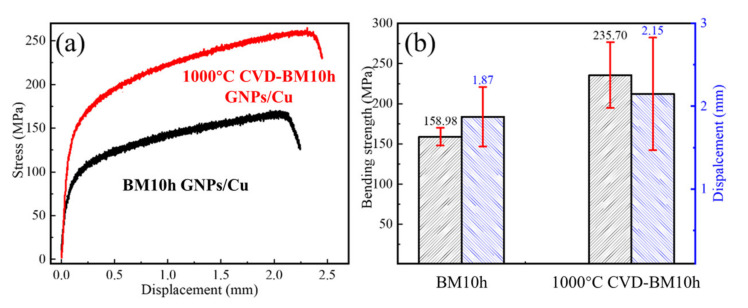
Bending performance of composites made by BM10h and 1000 °C CVD-BM10h. (**a**) Deflection-stress curve, (**b**) Flexural strength and fracture deflection of composite.

Afterward, the composite material was tested for bending performance, and the results are shown in Figure 13. It can be seen that the bending strength of the GNPs/Cu composite after CVD repair has been increased from 158.98 MPa to 235.70 MPa, an increase of 48%. The fracture deflection has been increased from 1.87 mm to 2.15 mm, an increase of 14%, and the strength and plasticity have been improved at the same time.

**Figure 14:**
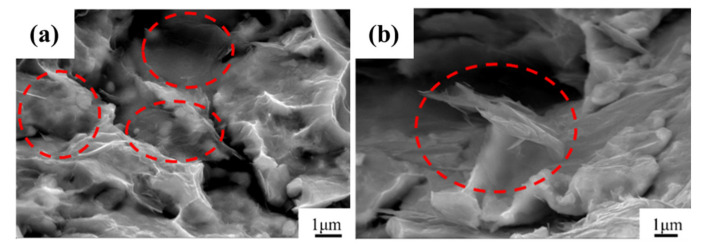
Fracture morphology of composites. (**a**) Fracture analysis of composites made by BM10h, (**b**) Fracture analysis of composites made by 1000 °C CVD-BM10h.

The fracture of the composite is observed as shown in Figure 14, and graphene can be observed at the fracture. Graphene is mostly thin and has better electron beam penetration, and the Cu matrix underneath can be observed during imaging. It can be seen that the fracture of the composite material shows a more obvious layered morphology, and the layered morphology after the CVD process is more obvious than that of the composite material without CVD treatment. This is because in the CVD process, the high temperature causes the sintering phenomenon between the Cu particles, which makes the originally smaller Cu flakes connected into Cu flakes with larger particle size. Since the powder does not melt during the SPS process, this large layered shape is retained.

**Figure 15:**
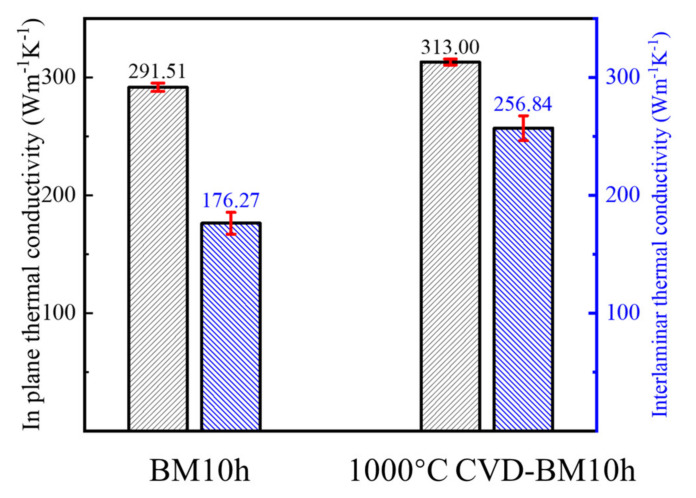
Thermal conductivity characterization results of composites made by BM10h and 1000 °C CVD-BM10h.

The thermal conductivity of the two composites was characterized, the temperature used in the test was normal, and the results are as shown in Figure 15. It can be seen from the results that the in-plane and inter-plane thermal conductivity of the composite material prepared by the powder repaired by the 1000 °C CVD process has been improved. It shows that after the repair process, the defect content of the graphene is reduced and the thermal conductivity is improved, making the composite material obtain a higher thermal conductivity.

## 4. Conclusions

In this paper, the evolution and control of defects in graphene processing are studied. Through mechanical ball milling, the damage behavior of graphene during ball milling was studied, and the relationship between ball milling time and graphene defect content was established. The graphene defects were repaired by CVD, and the effects of temperature and initial defect content on the graphene repair process were studied.

Studies have found that CVD has a repairing effect on graphene defects, which can be significantly observed before and after the repair of graphene samples after ball milling for 5–10 h. The more the defect content, the more obvious the CVD repair effect. The defects of graphene ball milled for 10 h decreased significantly with the increase of CVD temperature, reflecting the promotion of temperature on the graphene repair process. Combined with Raman and XPS, the evolution mechanism of defects is analyzed. Since CVD repair is a process of carbon source gas cracking to generate active C atoms, which are deposited on the surface of graphene. Simultaneously existing in the CVD process: graphene repair, decomposition of oxygen-containing functional groups, and at the same time, with excess C atoms, new graphene with more defect content is generated in situ on the Cu matrix. When the CVD temperature is low, the deposition of C atoms dominates, and a large amount of new graphene with defects is deposited on the surface of the original GNPs-Cu, which leads to an abnormal increase in the content of defects. Increasing the CVD temperature will greatly increase the diffusion rate of C atoms, and the hole defects will be repaired by C atoms. The evolution of defects in graphene during CVD is the result of the coordination of three functions.

On this basis, the mechanical properties and thermal conductivity of composite materials prepared by CVD repaired graphene were studied, and it was found that the bending strength was increased by 48%, and the plasticity was also slightly improved. After the repair, the thermal conductivity also has a significant increase in the in-plane and inter-layer thermal conductivity, which is mainly attributed to the repair of defects by CVD.

## Data Availability

The data presented in this study are available on request from the corresponding author.
